# Galectin from *Trichinella spiralis* alleviates DSS-induced colitis in mice by regulating the intestinal microbiota

**DOI:** 10.1186/s13567-023-01262-x

**Published:** 2024-01-03

**Authors:** Jianqing Li, Xiangjiang Wang, Qiuhui Wang, Yishen Hu, Shouan Wang, Jia Xu, Jianbin Ye

**Affiliations:** 1 School of Pharmacy, Fujian Medical University, Fuzhou, 350004 Fujian China; 2 School of Basic Medicine Science, Putian University, Key Laboratory of Translational Tumor Medicine in Fujian Province, Putian, 351100 Fujian China; 3 School of Pharmacy, Putian University, Putian, 351100 Fujian China

**Keywords:** *Trichinella spiralis*, inflammatory bowel disease, galectin, microbiota

## Abstract

According to numerous reports, *Trichinella spiralis* (*T. spiralis*) and its antigens can reduce intestinal inflammation by modulating regulatory immunological responses in the host to maintain immune homeostasis. Galectin has been identified as a protein that is produced by *T. spiralis*, and its characterization revealed this protein has possible immune regulatory activity. However, whether recombinant *T. spiralis* galectin (*rTs*-gal) can cure dextran sulfate sodium (DSS)-induced colitis remains unknown. Here, the ability of *rTs*-gal to ameliorate experimental colitis in mice with inflammatory bowel disease (IBD) as well as the potential underlying mechanism were investigated. The disease activity index (DAI), colon shortening, inflammatory cell infiltration, and histological damage were used as indicators to monitor clinical symptoms of colitis. The results revealed that the administration of *rTs*-gal ameliorated these symptoms. According to Western blotting and ELISA results, *rTs*-gal may suppress the excessive inflammatory response-mediated induction of TLR4, MyD88, and NF-κB expression in the colon. Mice with colitis exhibit disruptions in the gut flora, including an increase in gram-negative bacteria, which in turn can result in increased lipopolysaccharide (LPS) production. However, injection of *rTs*-gal may inhibit changes in the gut microbiota, for example, by reducing the prevalence of *Helicobacter* and *Bacteroides*, which produce LPS. The findings of the present study revealed that *rTs*-gal may inhibit signalling pathways that involve enteric bacteria-derived LPS, TLR4, and NF-κB in mice with DSS-induced colitis and attenuate DSS-induced colitis in animals by modulating the gut microbiota. These findings shed additional light on the immunological processes underlying the beneficial effects of helminth-derived proteins in medicine.

**Supplementary Information:**

The online version contains supplementary material available at 10.1186/s13567-023-01262-x.

## Introduction

Inflammatory bowel disease (IBD), which includes ulcerative colitis (UC) and Crohn’s disease (CD), is a chronic and recurrent autoimmune condition that affects the gastrointestinal tract. Due to changes in lifestyles and living conditions, the prevalence of IBD has increased over the past few decades [1, 2]. IBD patients experience emotional stress and financial strain due to the clinical manifestations of the disease, which include diarrhoea, bloody mucopurulent stools, and abdominal pain [2, 3]. The molecular pathogenesis of IBD is unknown, but it is thought to be influenced by the host immune system, environmental infections, genetic predispositions, and the intestinal microbiota [4, 5]. Among these factors, it has long been hypothesized that the interaction of the gut microbiota with the mucosal immune system plays a role in the pathogenesis of IBD [5, 6].

Increasing evidence has emerged in recent years to support the idea that changes in the gut microbiota play a substantial role in the pathophysiology of IBD [6, 7]. Patients with IBD frequently exhibit dysregulation of the intestinal mucosal barrier as well as a decrease in microbial diversity and disruption of the gut microbiota [8]. Numerous studies have reported changes in the proportions of “commensal” and “harmful” bacteria in the intestines of IBD patients, including a decrease in the abundance of Firmicutes and Bacteroidetes and an increase in the abundance of Proteobacteria [9, 10]. Accordingly, studies have shown that probiotic therapy and faecal bacteria transplantation are two effective therapeutic options for treating intestinal microbiota dysbiosis in IBD patients [11–13].

Interestingly, improved hygiene and decreased rates of infection with intestinal helminths and other organisms have been linked to the increase in IBD cases [14]; this relationship is known as the “hygiene hypothesis”. A lack of exposure to antigens, such as those derived from helminths or other parasites, in the early years of life increases the risk of developing allergies and autoimmune diseases, such as IBD. For instance, helminth infection is necessary for intestinal immunological homeostasis [15, 16]. As a result, it appears that the prevalence of IBD and the rate of helminth infection are inversely associated [17]. The ability of helminths to control the host immune system may also explain this difference [18, 19]. Prior research has demonstrated that excretory-secretory products play crucial roles in regulating the immune response by exerting anti-inflammatory effects, [20, 21]. Thus, helminthic therapy is a potential method for treating IBD. However, helminthic therapy can result in parasitic infection, which raises biosafety concerns. Thus, according to various investigations, proteins that are extracted from worms could be used to treat IBD as an alternative to parasite infestation [17, 19, 21–25].

*Trichinella spiralis* (*T. spiralis*) is a parasitic zoonotic worm that infects a variety of vertebrate hosts, including humans, and it is a unique modulator of inflammatory responses [26]. The consumption of raw meat that is contaminated with *T. spiralis* larvae frequently results in human disease. Muscle larvae (ML), which have already infected the host, are released into the stomach and subsequently mature into intestine-infective larvae (IIL). The IIL penetrate the intestinal epithelium and develop into adult worms (AWs) after four moults. Female adults shed new-born larvae (NBL), which travel throughout the body via the circulatory system to reach skeletal muscles, where they ultimately parasitize and develop into ML [27]. Through the release of excretory-secretory (ES) chemicals, ML convert muscle cells into encapsulated nurse cells without killing them, ensuring their long-term survival and immunological crosstalk with the host [26]. Several studies have demonstrated that *T. spiralis* infection can ameliorate immune-mediated diseases, including IBD, allergies, and rheumatoid arthritis (RA). In a previous study, *T. spiralis* excretory-secretory antigens were shown to alleviate experimental autoimmune encephalomyelitis by stimulating dendritic cells [28]. According to other studies, the immune-regulatory effects of ES products from *T. spiralis* on macrophages and Tregs may be responsible for the observed decrease in colonic inflammation [21, 29]. Thus, the ES products of *T. spiralis* play key roles in the immune crosstalk between *T. spiralis* and the host, and these roles could be related to the pathways that regulate inflammation in the intestine.

There have been numerous reports of the expression of galectins, a family of lectins that bind to galactosides, by many parasites [30, 31], including *T. spiralis*. To date, galectins have been classified into 15 different types, including 9 prototypes (galectins 1, 2, 5, 7, 10, 11, 13, 14, and 15), 5 TR types (galectins 4, 6, 8, 9, and 12), and only 1 chimaera type (galectin 3) [32]. Galectins can influence a wide range of cellular and intracellular processes, including fibrosis, organogenesis, immune responses, and malignancy, making them viable therapeutic targets for inflammatory diseases [33]. Numerous investigations have described the connection between host immunomodulation and the galectins of parasites. For instance, in the autoimmune encephalomyelitis (EAE) model, galectins from *Toxascaris leonine* might increase EAE severity and antibody production [34]; however, they exert a positive effect on the IBD model by considerably increasing the levels of TGF-β and IL-10 [35]. Moreover, galectin from *Haemonchus contortus* (*H. contortus*) is a promising vaccine target for preventing *H. contortus* infection [31]. One galectin from *T. spiralis* was isolated, described, and shown to enhance larval invasion of host intestinal epithelial cells in our previous investigation [30]. However, it is still unclear whether *T. spiralis* galectin has a beneficial or negative effect on the regulation of the host immune system.

We hypothesize that *T. spiralis* galectin may also perform some immunomodulatory functions in the host immune system given the substantial evidence demonstrating that ES products from *T. spiralis* are able to ameliorate IBD and that galectin potentially functions in modulating autoimmune diseases. In this study, *rTs*-gal was injected into mice with DSS-induced colitis to treat these model mice. Changes in the gut microbiota were subsequently investigated to determine the effect of immunization with *rTs*-gal on the IBD model.

## Materials and methods

### Materials, animals and parasites

DSS (MW 36 000–50 000 Da) was purchased from a commercial company (MP Biomedicals, LLC, Santa Ana, CA, USA), and anti-TLR4, anti-TLR2, anti-β-actin, anti-MyD88, and anti-NF-κB antibodies were purchased from ImmunoWay Biotechnology (5048 Tennyson Pkwy Ste 250, Plano, TX,75024 USA). ELISA kits for measuring cytokine concentrations were purchased from Novoprotein (Suzhou, Jiangsu Province, China). Male BALB/c mice aged 6–8 weeks were purchased from Wushi Animal Center (Fuzhou, Fujian Province, China). The *T. spiralis* strain (ISS534) was maintained in our department by passage in mice. The animal experiments in this study were approved by the Life Science Ethics Committee of Putian University (No. 2021 (6)), and efforts were made to minimize the number of animals used and their suffering.

### Preparation of *rTs*-gal and haemagglutination activity analysis

Recombinant *T. spiralis* galectin (XP003381656.1) was expressed in the *Escherichia coli* BL21 (DE3) system according to previously described methods [30]. *rTs*-gal was purified using Ni–NTA-Sefinose resin (Sangon Biotech Co. in Shanghai, China) and subjected to SDS‒PAGE. Haemagglutination was used in this study to assess *rTs*-gal activity. Blood samples were collected from mice, and erythrocytes were then washed three times in sterile saline solution (pH 7.0) after centrifugation at 350 × *g* for 10 min. The erythrocytes were resuspended in 2% saline solution. Then, haemagglutination activity assays were conducted as described in published papers with some modification [36, 37]. First, 25 μL of 2% erythrocyte suspension was applied to slides, followed by 25 μL of *rTs*-gal (100 μg/mL) or PBS. The slides were incubated at room temperature in a wet box for 1 h, and the outcome was then examined under a microscope.

### Establishment of the colitis model

**Figure 1:**
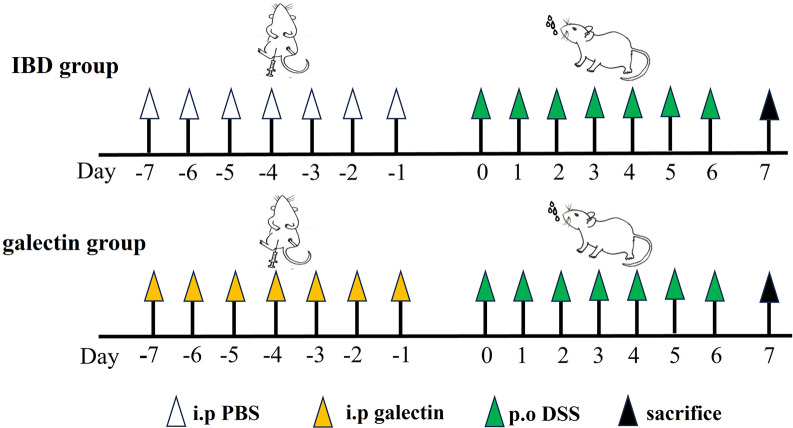
**Schematic diagram of colitis model establishment.** (*i.p* Injected intraperitoneally, *Po* Provides orally).

Ten mice were randomly assigned to one of the following three groups: (1) the control group (healthy mice were given sterile water alone); (2) the galectin group (mice were first treated with 20 μg of *rTs*-gal daily for 1 week and then challenged with 4% DSS daily for 1 week before being sacrificed on the last day); and (3) the IBD group (mice were treated as in the galectin group but treated with PBS instead of *rTs*-gal) (a schematic diagram of the experimental design is shown in Figure 1). According to the methods that were described a prior study [38], the body weight and DAI, which is determined based on body weight loss, stool consistency, and blood in the faeces, of the mice were assessed daily. Blood in mouse faeces was detected using the benzidine technique. One drop of prepared orthotoluidine solution and one drop of 3% hydrogen peroxide solution were added to mouse faeces that had been coated on a white porcelain plate. The amount of blood in the stool was recording according to the following colour changes: (1) the colour changed to dark green immediately (+ + +); (2) the colour turned blue after 30 to 60 s ( +); and (3) the colour remained the same for two minutes (-). On the seventh day after the model was established, all the mice were sacrificed, and samples were harvested for various tests. After the lengths of the mouse colons were measured, samples of the colon and faeces were preserved at −80 °C until use.

### Histological analysis

The colon was divided into two segments, namely, the proximal and distal colon. The distal colon was preserved using 10% formaldehyde and stained with haematoxylin and eosin according to previously published methods [39, 40]. HE staining was performed, and images were captured with an optical biological microscope. Scores were recorded for the following categories: epithelium loss, crypt damage, goblet cell depletion, and inflammatory cell infiltration. PAS staining was also performed, and the stained samples were observed under a microscope. Immunohistochemical (IHC) analysis of cytokines (IL-6, IL-1β, and TNF-α) in colon tissues was also carried out to investigate their differential expression, and their differential expression was investigated using Image-Pro Plus software.

### Western blotting analysis of TLR4 (TLR2)/MyD88/NF-κB expression in colon tissues

Colon tissues were cut and homogenized in lysis solution using a glass homogenizer. The samples were centrifuged at 14 000 × *g* for 5 min after complete lysis to extract the supernatants. The protein concentrations of the supernatants were measured using a bicinchoninic acid (BCA) protein assay kit (Sangon Biotech, Shanghai, China). The samples were separated using SDS‒PAGE and then blotted onto polyvinylidene difluoride (PVDF) membranes for 1 h at 375 mA using a Mini Trans-Blot® Cell (Bio-Rad, China). The membranes were incubated for 2 h at room temperature in blocking buffer (5% skim milk in TBST). After washing with TBST three times, the membranes were incubated with anti-TLR4 (TLR2), anti-NF-κB, anti-β-actin, and anti-MyD88 (diluted 1:1000 in TBST) antibodies at 37 °C for 1 h. The membranes were then treated with HRP-conjugated goat anti-rabbit IgG (1:5000) at 37 °C for 1 h after being washed with TBST three more times. After the final three washes, the membranes were stained with ultrasensitive ECL chemiluminescence reagent (Sangon Biotech, Shanghai, China), and the bands were quantified via densitometry and analysed via ImageJ.

### ELISA analysis

The levels of the cytokines IL-6, IL-1β, and TNF-α were evaluated by ELISA kits according to the manufacturer’s instructions. Briefly, blood samples were centrifuged at 3000 rpm/min for 15 min, the serum was separated, and the cytokine contents in the serum were measured.

### Statistical analysis

The data were statistically analysed using SPSS version 26.0. The data are presented as the mean ± SD of at least three repeated experiments. The data from different groups were evaluated using one-way analysis of variance (ANOVA). *P* < 0.05 was considered to indicate statistical significance.

### Analyses of the caecal microbiota

Total DNA was extracted from faecal samples that were collected from mouse colons (five samples in each group) using an E.Z.N.A. soil DNA kit (Omega Biotek, Norcross, GA, USA) according to the manufacturer’s instructions. A Nanodrop One UV‒vis spectrophotometer (Thermo Scientific) was used to measure the quantity and quality of the DNA. The V3-V4 region of the bacterial 16S rRNA gene was amplified using the universal primers 338F and 806R. The PCR parameters were chosen according to previous investigations [41]. The PCR products were purified using the AxyPrep DNA Gel Extraction Kit from Axygen Biosciences in Union City, California, and their quantity was determined using the QuantusTM Fluorometer from Promega in the United States. Using the MiSeq Reagent Kit v3, the purified amplicons were run on an Illumina MiSeq platform (Illumina, San Diego, USA) according to the recommended protocols by a for-profit organization (Majorbio Bio-Pharm Technology Co. Ltd., Shanghai, China). The NCBI Sequence Read Archive (SRA) database was used to obtain the raw sequence data (accession number: PRJNA1025354).

The Majorbio Cloud Platform’s online tool was used for data processing. Briefly, the primer sequences were removed from the clean reads using Fastp version 0.20.0 after the clean reads were first assembled using FLASH version 1.2.11. After the putative chimeric sequences were filtered, sequencing reads with exact matches to barcodes were recognized as authentic sequences and assigned to the correct samples. Operational taxonomic units (OTUs) with a 97% similarity threshold were grouped using UPARSE version 7.1. Using the RDP Classifier version 2.2, all the representative reads were annotated using the Silva database Version 138 (16S rRNA) with a confidence level of 80%.

Mothur version 1.30.1 was used to examine the alpha diversity of the microbiota. The Bray‒Curtis dissimilarity was used to perform principal component analysis (PCA) and nonmetric multidimensional scaling (NMDS) to highlight the differences among the three groups. Analysis of similarity (ANOSIM) was used to examine the differences between groups. To elucidate the distances among all the samples, a hierarchical clustering tree was constructed for the genera, and a heatmap was generated. KEGG pathway analysis was used to examine the functional gene composition of the microbiota, and the distinctions between each group were also examined. A *P* value of less than 0.05 was used to determine the statistical significance of each finding.

## Results

### Preparation of *rTs*-gal and haemagglutination activity assay

**Figure 2:**
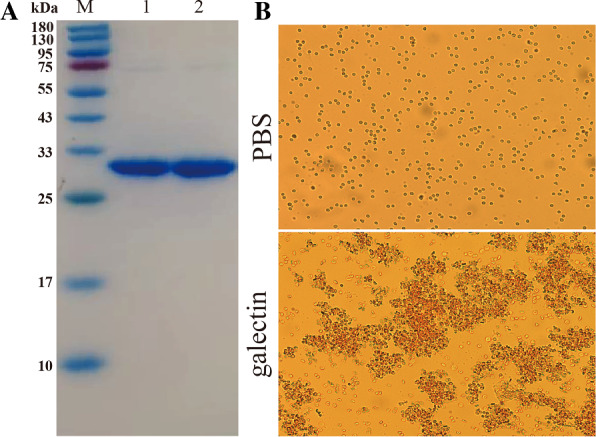
**SDS‒PAGE analysis of rTs-gal** **and haemagglutination activity assay.** **A **SDA-PAGE (M: marker; Lane 1 and Lane 2: replicates of purified rTs-gal). **B** Haemagglutinating activity assay.

Using a seamless cloning kit, a fragment (795 bp) of *T. spiralis* galectin was acquired, cloned, and inserted into the pQE-80L expression vector. The *rTs*-gal fusion protein was successfully expressed by *E. coli* BL21 harbouring pQE-80L/*Ts*-gal after IPTG induction. The protein was purified by utilizing a Ni–NTA-Sepharose column and is shown in Figure 2A. The haemagglutination activity results demonstrated the capacity of *rTs*-gal to agglutinate erythrocytes (Figure 2B), and this positive result suggested that the structure of *rTs*-gal was consistent with that of natural *Trichinella* galectin. The haemagglutinating activity of *rTs*-gal suggested that *rTs*-gal had been successfully prepared.

### *rTs-gal* ameliorates DSS-induced acute colitis

**Figure 3:**
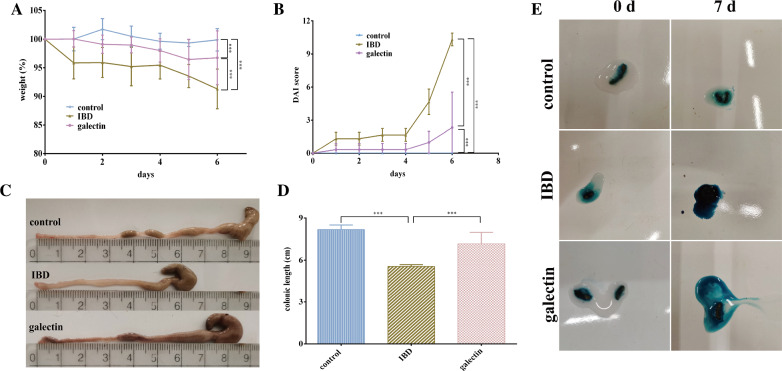
**Effects of rTs-gal on the pathological symptoms of DSS-induced acute colitis. A** Changes in body weight. **B** Disease activity index. **C** Representative colons from each group. **D** Colon length of each group. **E** Analysis of blood in faeces (faeces from the IBD group immediately became dark green). Significant differences are indicated by *P* < 0.05 (*), *P* < 0.01 (**), and *P* < 0.001 (***).

Mice were intraperitoneally administered 20 μg of *rTs*-gal daily for one week prior to DSS administration to assess the potential preventive effects of *rTs*-gal on IBD. After DSS induction, the mice with DSS-induced colitis (IBD group) displayed typical clinical colitis symptoms, including considerable weight loss and diarrhoea with bloody stools (Figures 3A and E). In addition, the colons of the IBD group were noticeably shorter than those of the other two groups (Figure 3C). In contrast, mice administered *rTs*-gal displayed less severe inflammation and had fewer clinical symptoms. Similarly, the DAI score of the galectin group (Figure 3B) was significantly (*P* < 0.01) lower than that of the IBD group. For instance, there was no difference (*P* > 0.05) in colon length between the galectin group and the control group (normal mice), and the body weight of the galectin group was also closer to that of the control group than that of the IBD group (Figures 3A and D).

**Figure 4:**
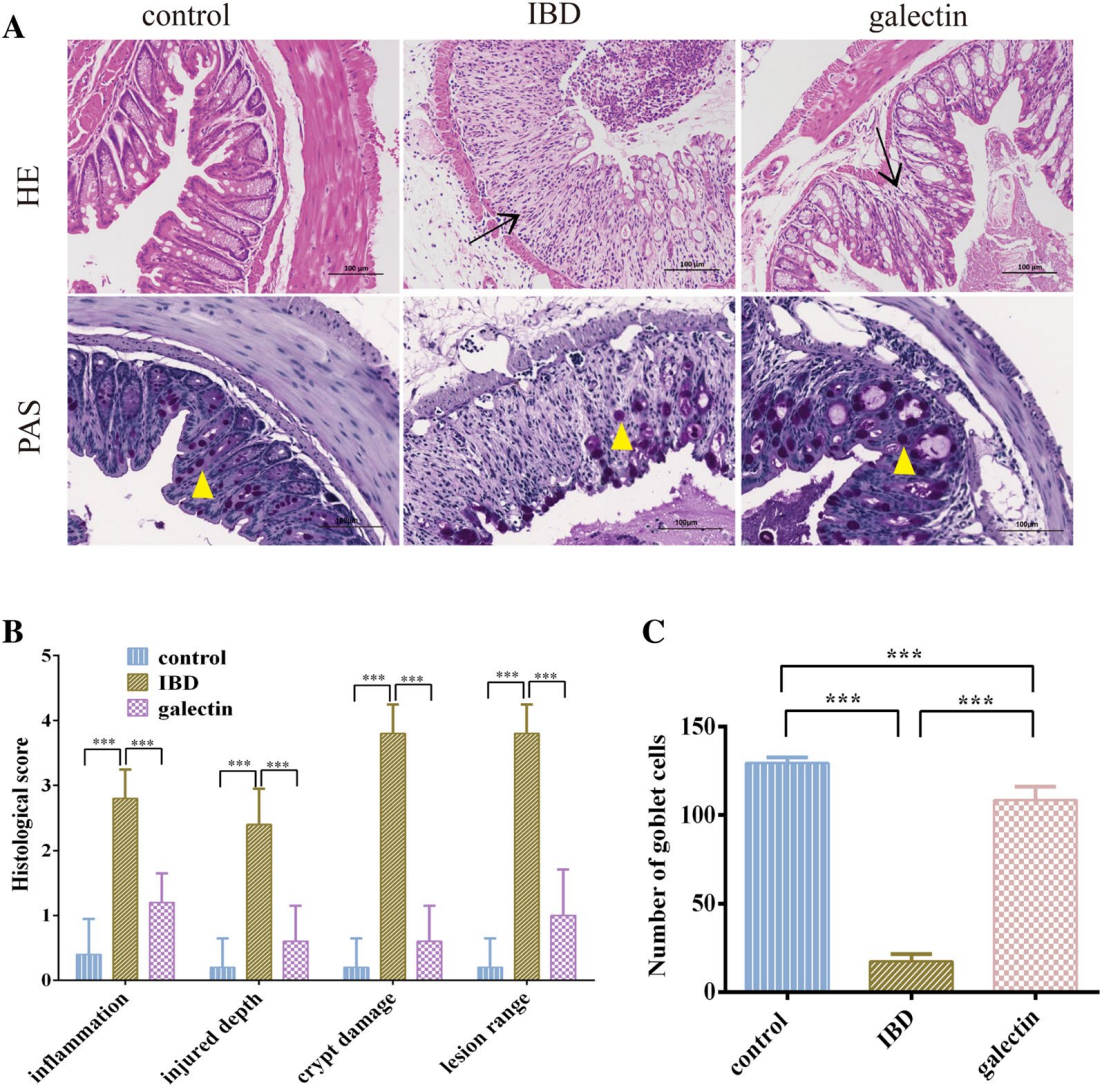
**Effects of rTs-gal on histopathological changes in DSS-induced acute colitis. A** Representative histopathological sections of the distal colon (bar: 100 µm, the arrows indicate inflammation, and the triangles indicate goblet cells). **B** Histological scores of representative colon tissues from each group. **C** The number of goblet cells. Significant differences were indicated by *P* < 0.05 (*), *P* < 0.01 (**), and *P* < 0.001 (***).

A histological study further showed that the administration of *rTs*-gal may treat DSS-induced acute colitis. H&E staining revealed that neither the galectin group nor the control group had any obvious evidence of inflammation (Figure 4A). However, the IBD group displayed significant crypt loss, inflammatory cell infiltration, and significant neutrophil infiltration. The degree of injury, crypt damage, lesion range, and infiltration were all dramatically reduced by the administration of *rTs*-gal (Figure 4B). Additionally, PAS staining revealed that the goblet and mucous coverage on the mucosal surface decreased to variable degrees in the IBD group (Figures 4A and C). However, in the galectin group, the atrophy of epithelial cells and goblet cells in the intestinal mucosa was reversed, and mucus secretion also increased.

### Effects of *rTs*-gal on the TLR4 (TLR2)/MyD88/NF-κB signalling pathway in DSS-induced model mice

**Figure 5:**
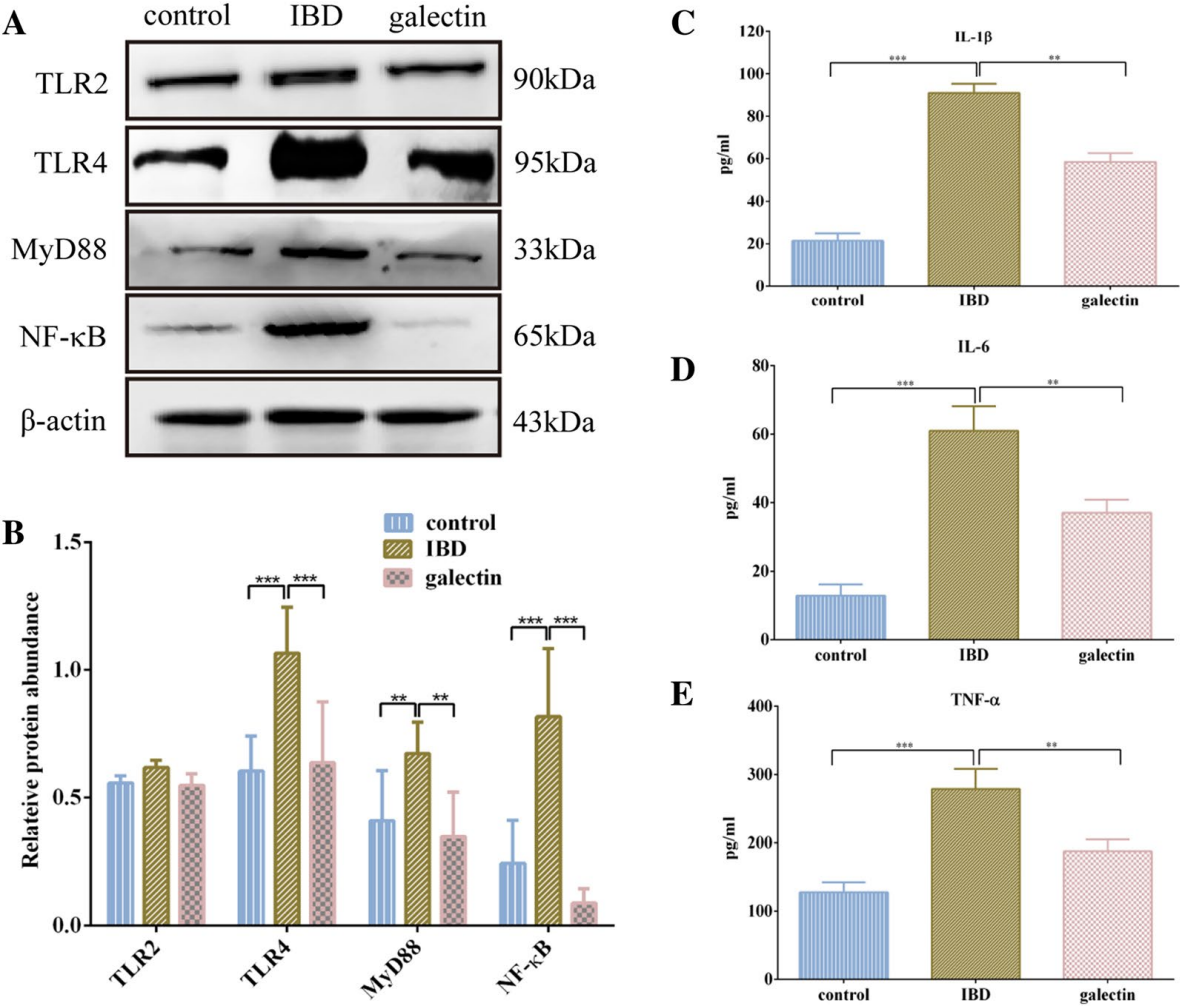
**Effects of rTs-gal on the expression of inflammatory mediators. A** Western blotting analysis of TLR2, TLR4, MyD88, and NF-κB expression. **B** Relative protein abundance was determined by densitometric analysis according to the Western blotting results, and β-actin was used as a reference. **C**, **D**, **E** ELISA analysis of cytokine levels (IL-1β, IL-6, TNF-α). Significant differences were indicated by *P* < 0.05 (*), *P* < 0.01 (**), and *P* < 0.001 (***).

The TLR signalling pathway is an important inflammatory signalling pathway that is involved in the development of IBD. Here, the expression of TLR2, TLR4, MyD88 and NF-κB in the colon was measured by Western blotting. The results showed that the expression of these proteins (excluding TLR2) was significantly higher in the IBD group than in the control group (Figures 5A and B), while the expression of both of these proteins was significantly downregulated by *rTs*-gal injection (*P* < 0.05). The production of proinflammatory cytokines, including IL-6, IL-1β, and TNF-α, may be promoted by activation of the TLR4/MyD88/NF-κB signalling pathway, exacerbating intestinal inflammation. The ELISA results revealed that the levels of these cytokines were significantly elevated in the IBD group, whereas treatment with *rTs*-gal reduced the levels of these cytokines (Figures 5C–E). This result was further confirmed by IHC analysis (Additional file 1).

### Effects of *rTs-gal* on microbial diversity

**Figure 6:**
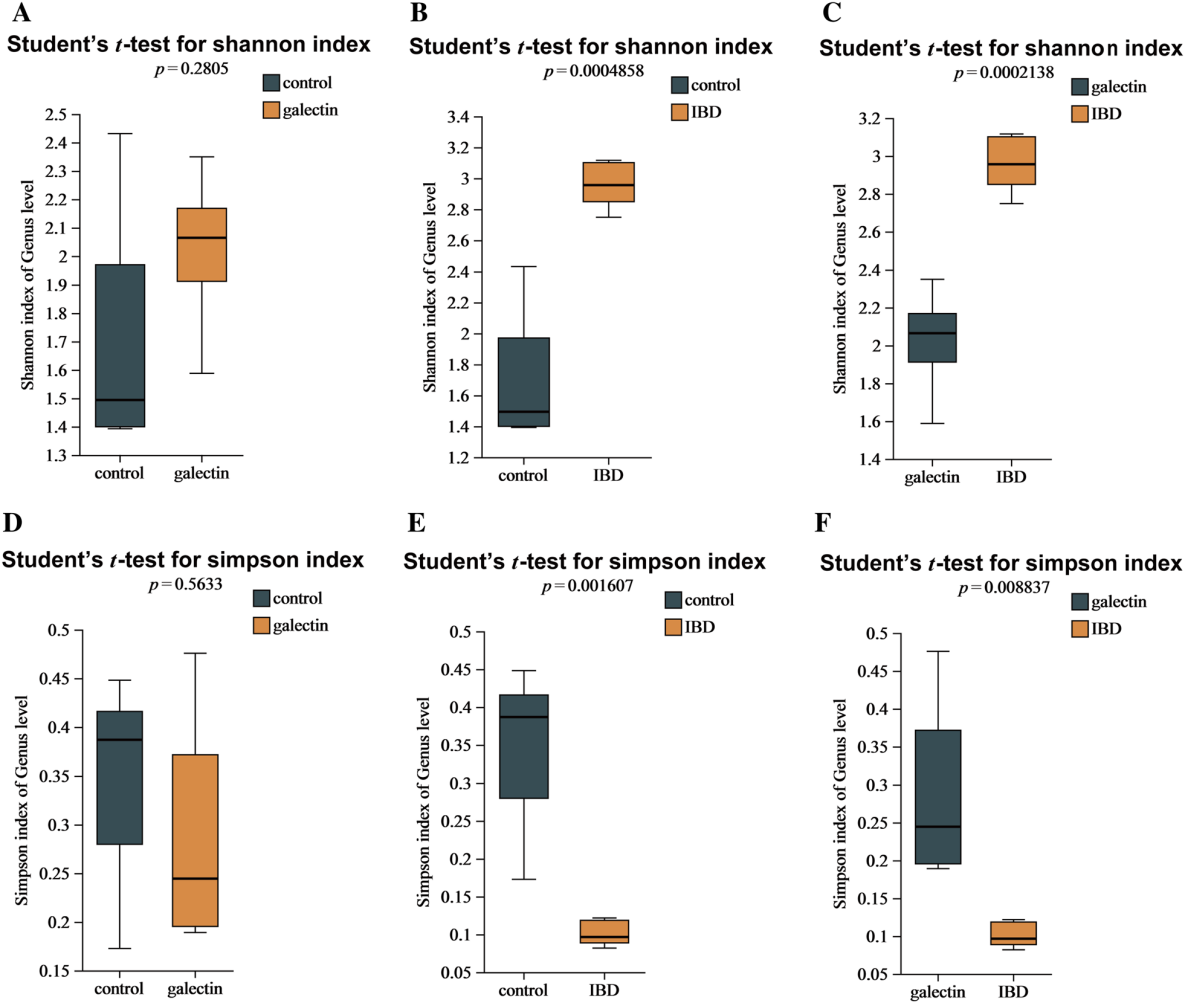
**The differences in α diversity indices between each group. A**, **B**, **C** Shannon index. **D**, **E**, **F** Simpson index.

To determine the potential impact of *rTs*-gal on the intestinal microbiota of mice treated with DSS, 16S rRNA sequencing was performed on faecal samples. In total, 15 faecal specimens from each of the three groups were sequenced, and a total of 6 717 636 sequences, or approximately 419 sequences per sample, were identified (Additional file 2). The rarefaction curve demonstrated that the sequencing results for all the samples plateaued, indicating that the depth was adequate (Additional file 3). Following analysis, the diversity was estimated to assess the richness and diversity of the microbiota (Figure 6). Interestingly, the diversity and quantity of the microflora were increased in the IBD group. The Shannon and Simpson indices showed that the diversity of the IBD group was significantly different from that of both the control group (*P* = 0.00048 for the Shannon index and 0.0016 for the Simpson index, < 0.01) and the galectin group (*P* = 0.00021 for the Shannon index and 0.0088 for the Simpson index, < 0.01). However, there were no discernible differences in microbial diversity between the galectin group and the control group (*P* = 0.5633 for the Simpson index and 0.2805 for the Shannon index, > 0.05). These findings suggested that *rTs-gal* injection might restore microbial diversity in DSS-induced model mice.

**Figure 7:**
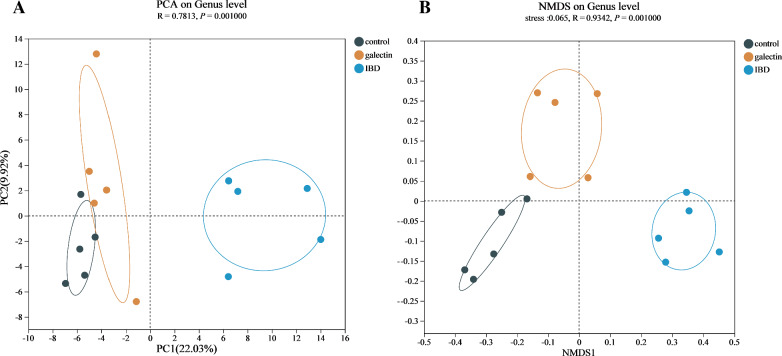
**The β diversity of all the groups at the genus level. A** PCA. **B** NMDS analysis.

**Figure 8:**
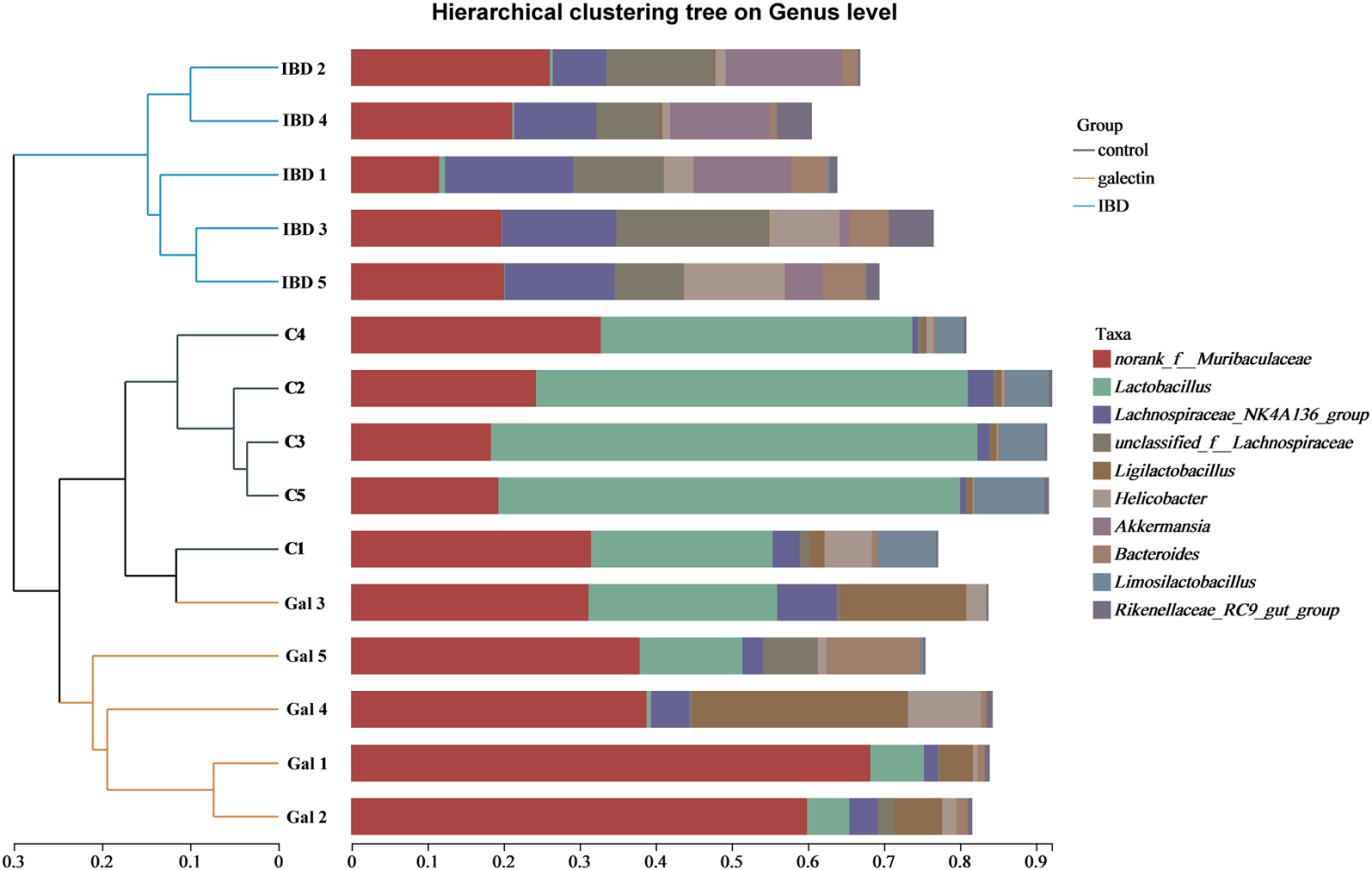
**Hierarchical clustering tree at the genus level for all samples.** The genera with a relative abundance < 0.05 were merged.

This finding was further supported by the beta diversity analyses that were conducted by PCA and NMDS. First, PCA (R = 0.7813, *P* = 0.001; ANOSIM) and NMDS (R = 0.3840, *P* = 0.001; ANOSIM) analyses demonstrated that the three groups could be distinguished from one another (Figure 7). Additionally, as shown by the heatmap of sample hierarchical clustering (Figure 8), all samples from each group could be effectively clustered based on the microbiota at the genus level. Treatment with *rTs*-gal was able to restore community diversity and shift the intestinal microbiota structure back in the direction of PC1, and a large distinct cluster between the IBD group and the control group was observed (Figure 7A).

### Effects of *rTs-gal* on the composition of bacterial communities

**Figure 9:**
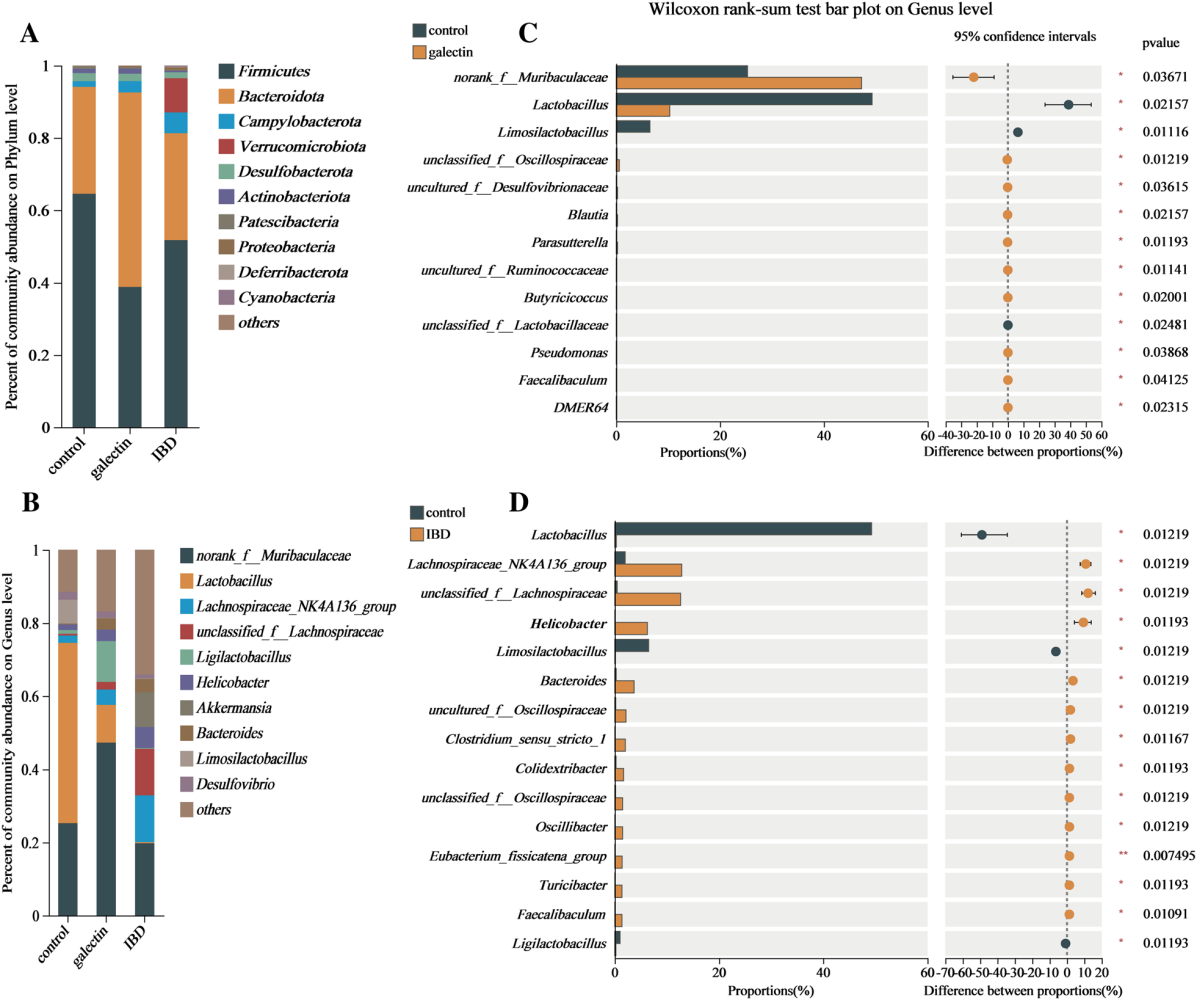
**The composition of the bacterial communities. A** Phylum level. **B** Genus level. **C** Significantly different genera between the control group and galectin group. **D** Significantly different genera between the control group and the IBD group.

To determine the specific impacts of *rTs*-gal on the intestinal flora, the composition of bacteria was examined at various levels (phyla and genus) (Figure 9 and Additional file 4). *Firmicutes* and *Bacteroidota* were the two bacterial taxa that dominated the bar plots for all three groups (Figure 9A). The IBD group had a somewhat lower abundance of *Firmicutes* (64.51% of the control group vs. 51.71% of the IBD group) and a higher abundance of *Campylobacterota* (1.55% of the control group vs. 5.79% of the IBD group) and *Verrucomicrobiota* (0.01% of the control group vs. 9.49% of the IBD group) than the control group. Compared to those in the control group, the abundances of *Firmicutes* (38.4%) and *Bacteroidota* (53.7%) were substantially higher in the galectin group. Although the change in the proportion of *Firmicutes* was not reversed by the administration of *rTs-gal*, the proportions of *Campylobacterota* (1.55% of the control group vs. 3.16% of the galectin group) and *Verrucomicrobiota* (0.01% of the control group vs. 0.01% of the galectin group) were similar between the galectin group and the control group, and the abundance of *Bacteroidota* was significantly greater in the galectin group than in the other two groups.

At the genus level (Figure 9B), the DSS-treated group exhibited a significant increase in the abundance of *Lachnospiraceae* (1.99% of the control group vs. 12.84% of the IBD group, *P* < 0.05), *Helicobacter* (1.55% of the control group vs. 5.76% of the IBD group, *P* < 0.05) and *Bacteroides* (0.27% of the control group vs. 3.72% of the IBD group, *P* < 0.05). Notably, *Lactobacillus* (49.28% of the control group) was the first dominant genus, whereas a clear decrease in *Lactobacillus* abundance (49.28% vs. 0.31%, *P* < 0.05) was observed in the IBD group. When *rTs*-gal was administered to DSS-induced model mice, the changes in the abundances of *Lachnospiracea*e (4.21%), *Helicobacter* (3.16%), *Bacteroides* (3.09%), and *Lactobacillus* (10.30%) were reversed to some extent. Moreover, *Muribaculaceae* (47.26% vs. 25.30%, *P* < 0.05) and *Ligilactobacillus* (11.12% vs. 1.01, *P* < 0.05) were obviously more abundant in the treatment group than in the control group (Figure 9D). All genera that exhibited significant differences between each group were also analysed by LEfSe (LDA score > 4.0, *P* < 0.05) and are shown in Additional file 5; the results were consistent with the bar plot.

**Figure 10:**
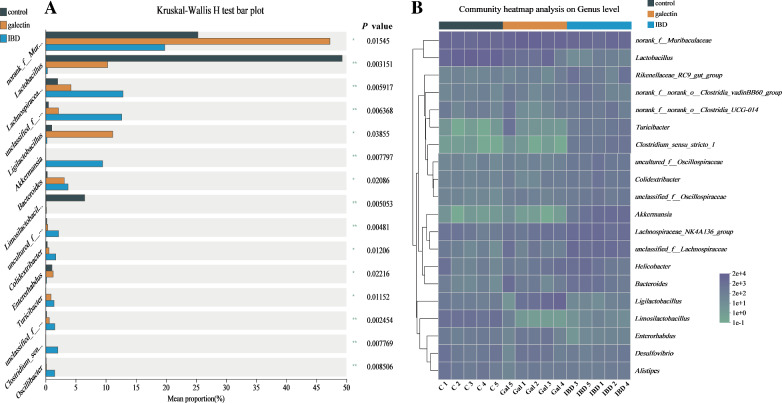
**Differential genera among the three groups (A) and heatmap of all the groups at the genus level (B).**

Furthermore, the differences in genera among the three groups were assessed to confirm these results. The results revealed fifteen genera that were significantly differently abundant, and their abundance in the galectin group was more similar to that in the control group than that in the IBD group (Figure 10A). A community heatmap analysis (Figure 10B) at the genus level (the top 20 genera with high abundance) also suggested that the microbiota communities in the mouse intestine were restored after treatment with *rTs-gal*. Thus, these results indicated that the administration of *rTs-gal* could reverse the disruption of microbiota communities in DSS-induced model mice.

### Effects of *rTs-gal* on the functional gene composition of the microbiota

**Figure 11:**
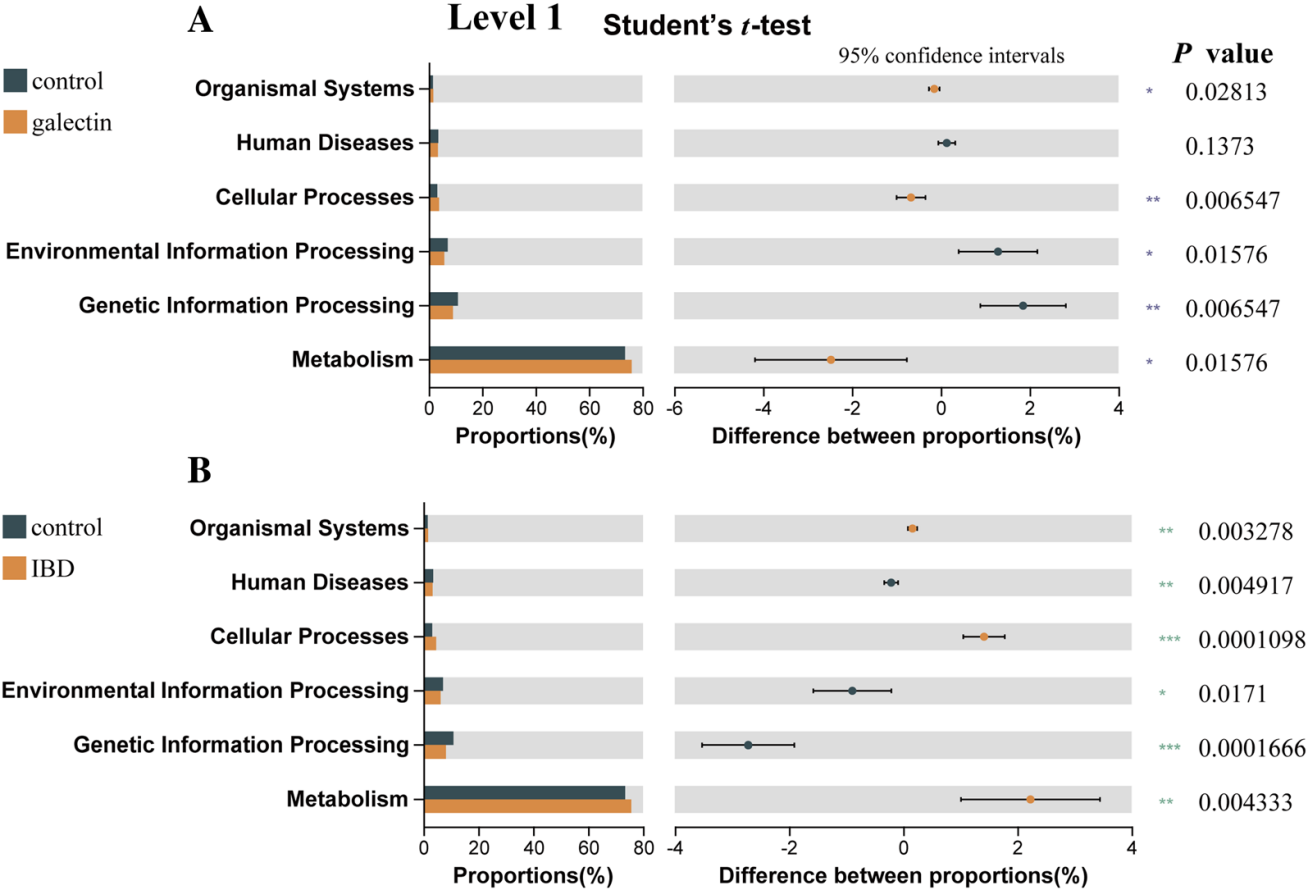
**Differences in functional capability analysis based on the KEGG pathways at Level 1. A** Differences between the control and galectin groups. **B** Differences between the control and IBD groups. Significant differences were indicated by *P* < 0.05 (*), *P* < 0.01 (**), and *P* < 0.001 (***).

**Figure 12:**
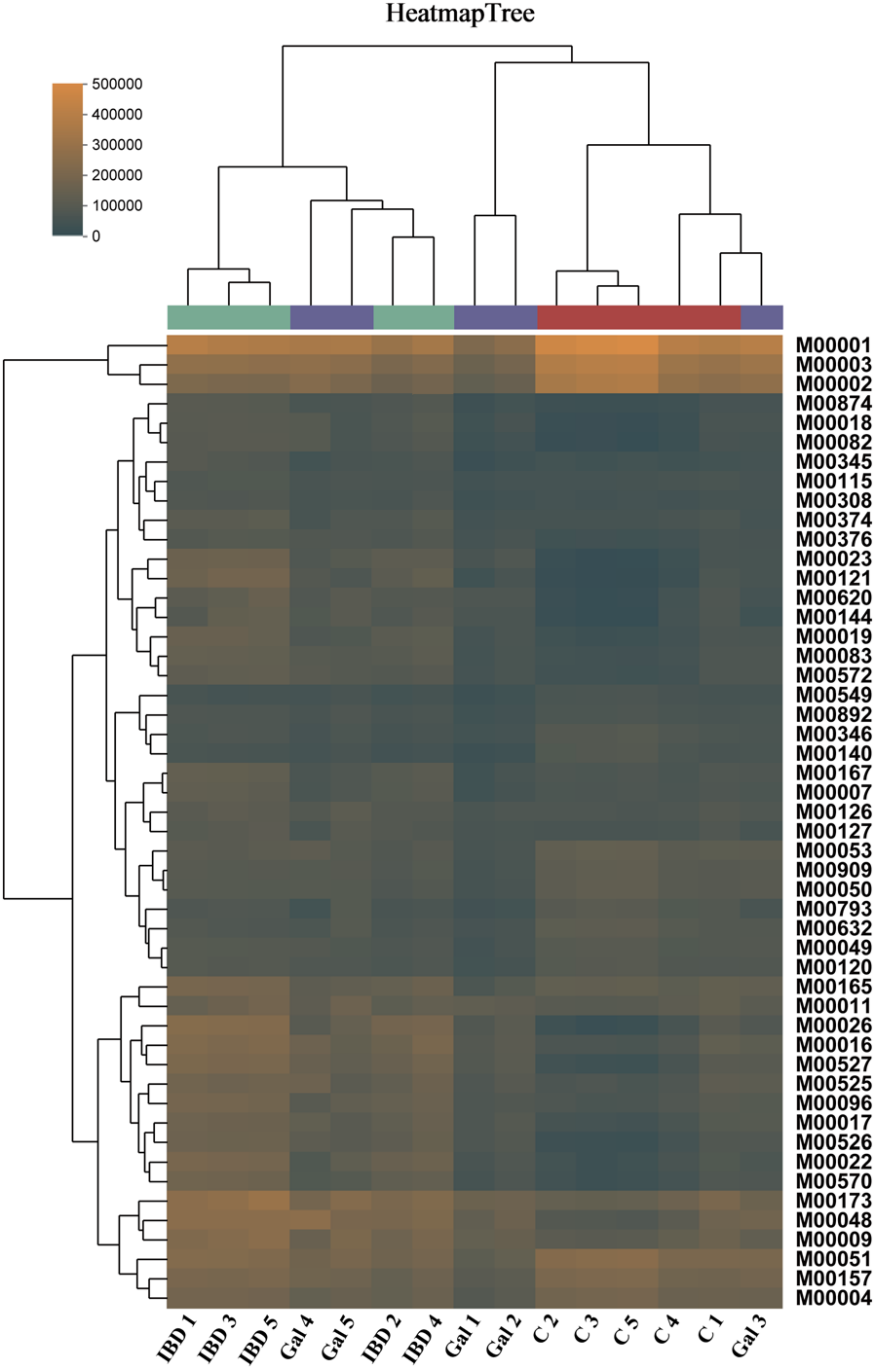
**Heatmap of the functional capability analysis of all the samples**.

Some of the possible biological functions in the intestinal tract may be represented by the functional gene composition of the microbiota. Using PICRUSt2 (Student’s *t* test), we also compared the functional gene compositions of all the groups. For all the samples combined, 263 KEGG level 3 modules were identified (Additional file 6). The results revealed that there were significant differences between the control group and the IBD group for each of the six KEGG pathways at level 1 (*P* < 0.01) (Figure 11B). Except for “human disease”, the majority of the KEGG pathways exhibited significant differences between the control group and galectin group, but the *P* values were lower (Figure 11A). Except for two samples in the galectin group (Gal 4 and Gal 5), the heatmap tree of the functional module clearly demonstrated that the majority of the samples from the galectin group were closely clustered with samples from the control group (Figure 12). All of the above results demonstrated that the administration of *rTs-gal* could ameliorate the changes in intestinal function in mice treated with DSS.

## Discussion

Chronic and recurrent intestinal immunological disorders, including IBD, still have unknown pathogenic causes. Importantly, IBD is associated with a significant risk of colon cancer development and has been associated with increased morbidity and death in recent years. Conventional treatments, such as anti-inflammatory or immunosuppressive medications, may cause some unwanted effects, including gastrointestinal problems, myocardial infarction, cancer, and blood‒brain barrier thrombosis [42]. As a result, prolonged usage of these medications is insufficient to satisfy the comprehensive therapeutic requirements of IBD patients.

According to recent reports, helminth infections and helminth-derived proteins are used in experimental immunotherapy and even in clinical trials for IBD [43, 44] or allergic illnesses [45, 46]. According to a prior study, “helminth therapy” for IBD involves the use of crude helminth extracts, refined compounds, and live ova/larvae [47]. However, the mechanics of this “helminth therapy” in IBD need additional research.

Galectin, a purified protein that is produced from *T. spiralis*, was administered to mice with DSS-induced colitis in the present study to ameliorate their symptoms. The use of *T. spiralis* and its antigens as a treatment for IBD has been frequently documented [48]. The galectin protein of helminths has been extensively reported, and our previous study [30] was the first to reveal its expression by *T. spiralis*. The results of this investigation revealed that galectin may have both therapeutic and preventive effects on DSS-induced colitis in mice. Injection of *rTs*-gal significantly slowed the onset of colitis in DSS-induced model mice by improving clinical presentation and reducing pathology in the inflamed colon.

The present study elucidated the mechanism by which *rTs*-gal treats colitis in mice. Several inflammatory cytokines play important roles in the onset and progression of the inflammatory response in IBD, and increased levels of these inflammatory cytokines in IBD patients can be considered a hallmark of disease [49]. One of the important inflammatory signalling mechanisms involved in the development of IBD is the TLR4 signalling pathway [50]. We used western blotting to measure TLR4, MyD88 and NF-κB expression, and the findings revealed that their expression levels were greater in the IBD group than in the control group, which was consistent with the results of other DSS-induced mouse models [51]. TLR4 and MyD88 expression decreased after delivery of *rTs*-gal, indicating a potential reduction in the inflammatory response. Previous research suggested that the paramyosin (*Ts*Pmy) produced by *T. spiralis* can stimulate regulatory T cells (Tregs) to preserve gut immunological homeostasis during inflammation [14]. Other research has also indicated that the *T. spiralis* serine protease may trigger a T helper type 2 (Th2)-type immune response and balance the trinitrobenzene sulfonic acid (TNBS)-induced Th1-type immune response [17] and that the effect of *T. spiralis* serine in relieving colitis is mediated by the activation of macrophages [22]. These findings suggested that numerous chemicals generated by *T. spiralis* might influence the gut immune system via multiple signalling pathways.

In recent years, increasing evidence has suggested that the gut microbiota plays a key role in regulating the pathogenesis of IBD [52]. Over 100 trillion bacteria have been identified in the digestive tract, and their biological activities are essential for preserving the stability of the intestinal immune environment [53, 54]. Studies have shown that intestinal inflammation in IBD patients can be caused by changes in the composition of the gut microbiota. IBD patients frequently exhibit significant dysbiosis of the gut flora, as evidenced by a decrease in *Firmicutes* and *Bacteroidetes* or an increase in *Proteobacteria* [52, 55]. Consistent with the findings of other studies, we also detected a lower abundance of *Firmicutes* in the IBD group in this study. Although the abundance of *Firmicutes* in the galectin group was also reduced, the abundance of *Bacteroidetes* was significantly increased. The increase in *Bacteroidetes* also suggested that the mechanism through which galectin mitigates IBD could involve regulating the intestinal microbiota.

Colitis in mice is primarily caused by an imbalance in the microbiota composition and the innate immune system [56]. According to certain studies, LPS from pathogenic bacteria may damage the intestinal barrier, decreasing the integrity of the epithelial mechanical barrier and ultimately leading to colitis [57]. Thus, regulating IBD-related intestinal flora dysbiosis is important for restoring intestinal inflammation. There is evidence that helminth-derived galectin can treat various immunological disorders [58], but there is no proof that it can control the intestinal microbiota. In this study, we discovered that *rTs*-gal could reverse the DSS-induced changes in the intestinal flora in mice. After the injection of *rTs*-gal, the microbial diversity (including α-diversity and β-diversity) revealed that the changes in intestinal flora caused by DSS were less pronounced.

Contrary to earlier studies [51, 54], the diversity was noticeably greater in the IBD group. At the genus level, *Lactobacillus* (accounting for 49.28%) was the dominant genus in the control group. The genus *Lactobacillus*, which belongs to the phylum *Firmicutes*, has been widely reported as a probiotic bacterium [59, 60]. These bacteria can interact with the gut epithelial lining to maintain the integrity of the gut barrier, protect the mucosal barrier, and treat DSS-induced colitis [61, 62]. These gut resident *Lactobacillus* strains can produce lactic acid, acetic acid, bacteriocins, and reactive oxygen species (ROS), which can all be used to kill pathogens directly. They can also play microbial roles by actively preventing opportunistic pathogens from occupying functional niches in the intestinal system [63]. Consequently, the extremely high relative abundance of *Lactobacillus* in the mice from the initial control group may be the cause of the low level of diversity. However, *Lactobacillus* was almost completely absent in the IBD group, which was associated with an increase in harmful bacteria (such as *Helicobacter*, *Lachnospiraceae* and *Bacteroides*). Nevertheless, the administration of r*Ts*-gal to the mice reduced the degree of dysbacteriosis, as the *Lactobacillus* abundance returned to 10.30%, and the relative abundance of harmful bacteria was also decreased compared with that in the IBD group. Notably, the abundance of *Muribaculaceae* and *Ligilactobacillus* was significantly increased in the galectin group, and these two genera have been demonstrated to be probiotic bacteria that can inhibit inflammation [64, 65]. These results suggested that the administration of r*Ts*-gal could alleviate colitis not only by reducing the DSS-induced structural changes in the intestinal flora but also by improving the abundance of some probiotic bacteria in the gut.

Inhibiting the proliferation of harmful bacteria can increase the relative abundance of useful bacteria in the gut microbiota. This can assist in reversing intestinal dysbacteriosis and restoring functional intestinal microecology. This finding was further confirmed by the analysis of the functional gene composition of the microbiota, which showed that the administration of r*Ts*-gal also alleviated possible changes in biological functions in the intestines of IBD model mice. In fact, LPS produced by gram-negative bacteria may cause the TLR4 signalling pathway to be activated in host cells. However, no significant differences in TLR2 expression were observed among the groups, which suggested that changes in the abundance of gram-positive bacteria in the gut may not be the reason for the alleviation of DSS-induced colitis in mice. An increase in TLR4 may subsequently cause MyD88 to become activated, which in turn promotes the production of inflammatory cytokines, including IL-6, TNF-α and IL-1β [66]. Dysfunction of the epithelial barrier may worsen as a result of these activations. The ELISA and Western blotting results in the present study supported these findings. The increase in the relative abundance of gram-negative bacteria (*Helicobacter* and *Bacteroides*) in the IBD group could corroborate these results, although we were unable to detect changes in the metabolites generated by the gut bacteria. Previous research has suggested that an imbalance in the species composition and metabolic state of the intestinal flora could contribute to the proliferation of dangerous bacteria such as *Bacteroides* [67]. Bacteroides can target the highly glycosylated polyprotein mucin in the mucus layer of the first protective layer of the intestinal epithelium, this promoting the host's intestinal inflammatory response. In the IBD group, *Helicobacter* was also more prevalent [68]. *Helicobacter* is a genus of gram-negative bacteria that are recognized to contribute to gastrointestinal human disease and that possesses a specific lipid A structure [69]. Taken together, these findings suggest that the administration of *rTs*-gal can treat DSS-induced ulcerative colitis by reducing the abundance of bacteria in the gut microbiota, particularly gram-negative bacteria. This decreases the synthesis of LPS in the intestinal environment, protecting the integrity of the intestinal barrier.

Overall, the findings of the present investigation suggested that treating DSS-induced ulcerative colitis with *rTs*-gal could be beneficial. The beneficial effects of *rTs*-gal on colitis may be achieved by controlling the intestinal flora and bacterial functions, which may reduce the generation of LPS and suppress the expression of TLR4, MyD88, and NF-κB. This process prevents the oversecretion of cytokines induced by the intestinal inflammatory response. This study provides new insight into the immunological mechanisms underlying the effect of helminth-derived proteins on alleviating IBD and reveals that *rTs*-gal may be a suitable therapeutic protein for IBD patients.

### Supplementary Information

**Additional file 1.** **Effects of *rTs-gal* on cytokines in colon tissues. (A) **Representative photomicrograph of cytokine expression as determined by IHC. **(B), (C)** and **(D)** Relative optical density of IL-1β, IL-6 and TNF-α, respectively.

**Additional file 2.** **Sequence information for all the samples. **Fifteen samples in three groups were sequenced, and the information (including sequence number, base number, mean length, minimum length, and maximum length) is provided in the table.

**Additional file 3.** **Rarefaction curves (Shannon indices) of all the samples.**

**Additional file 4.** **The detailed relative abundance of the bacterial community at the phylum and genus levels.** **(A) **and **(B)** Control group; **(C) **and **(D)** Galectin group; **(E) **and **(F)** IBD group.

**Additional file 5.** **LefSe analysis of all significantly different genera between each group.** Only genera with LDA scores >4.0 and *P *< 0.05 are listed in the figures.

**Additional file 6.** **The KEGG modules of all groups at the three levels.**

## Data Availability

The datasets used and/or analysed during the current study are available from the corresponding author upon reasonable request. The dataset supporting the conclusions of this article is included within the article.
