# Supplementary figures and images for: Galectin from Trichinella spiralis alleviates DSS-induced colitis in mice by regulating the intestinal microbiota
Source: Vet Res. 2024 Jan 3;55:3. doi: 10.1186/s13567-023-01262-x (PMC10763409; doi:10.1186/s13567-023-01262-x)

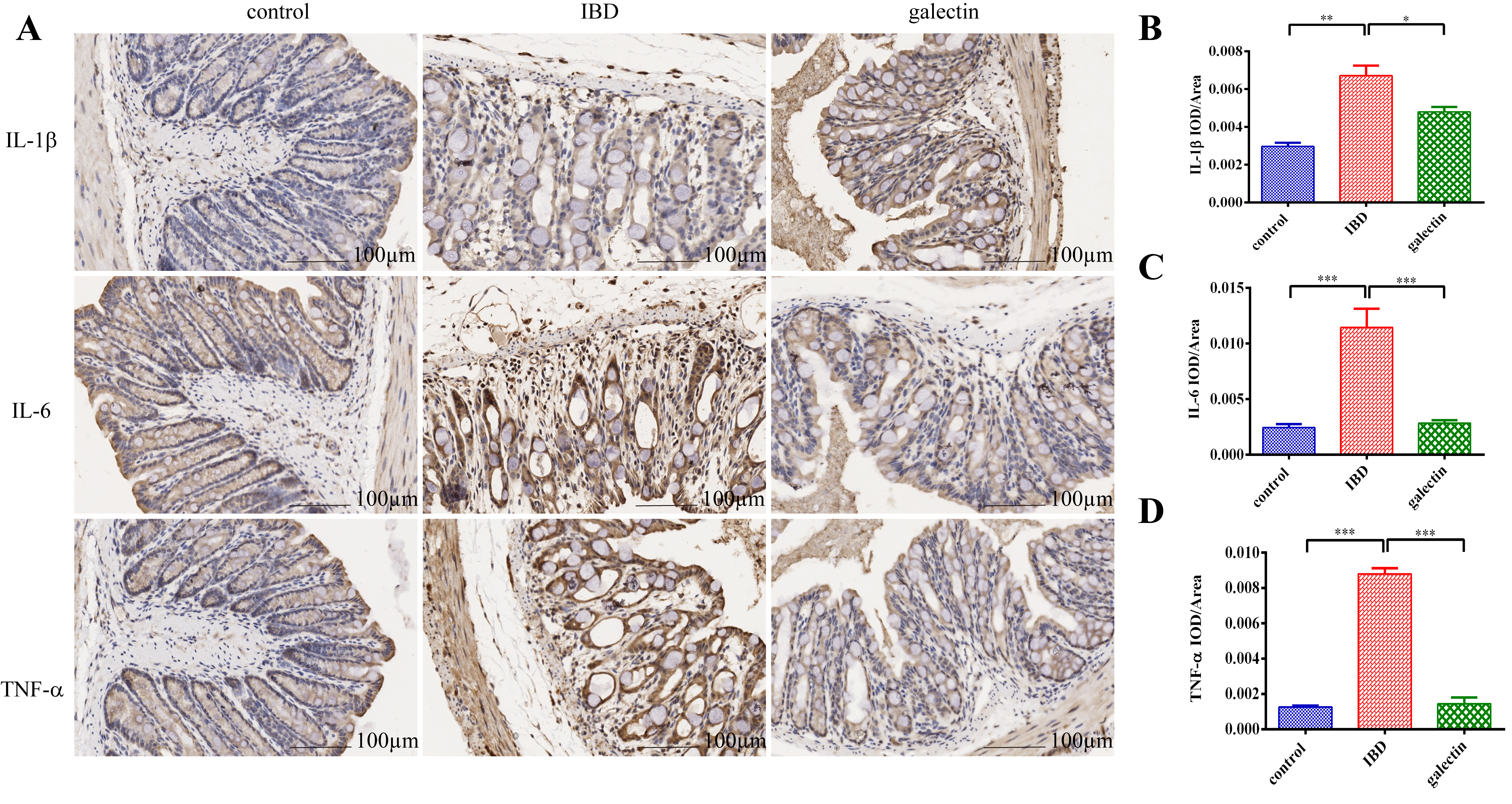

Supplement: Supplementary file 1 — Additional file 1. Effects of rTs-gal on cytokines in colon tissues. (A) Representative photomicrograph of cytokine expression as determined by IHC. (B), (C) and (D) Relative optical density of IL-1β, IL-6 and TNF-α, respectively. [file 13567_2023_1262_MOESM1_ESM.tiff]

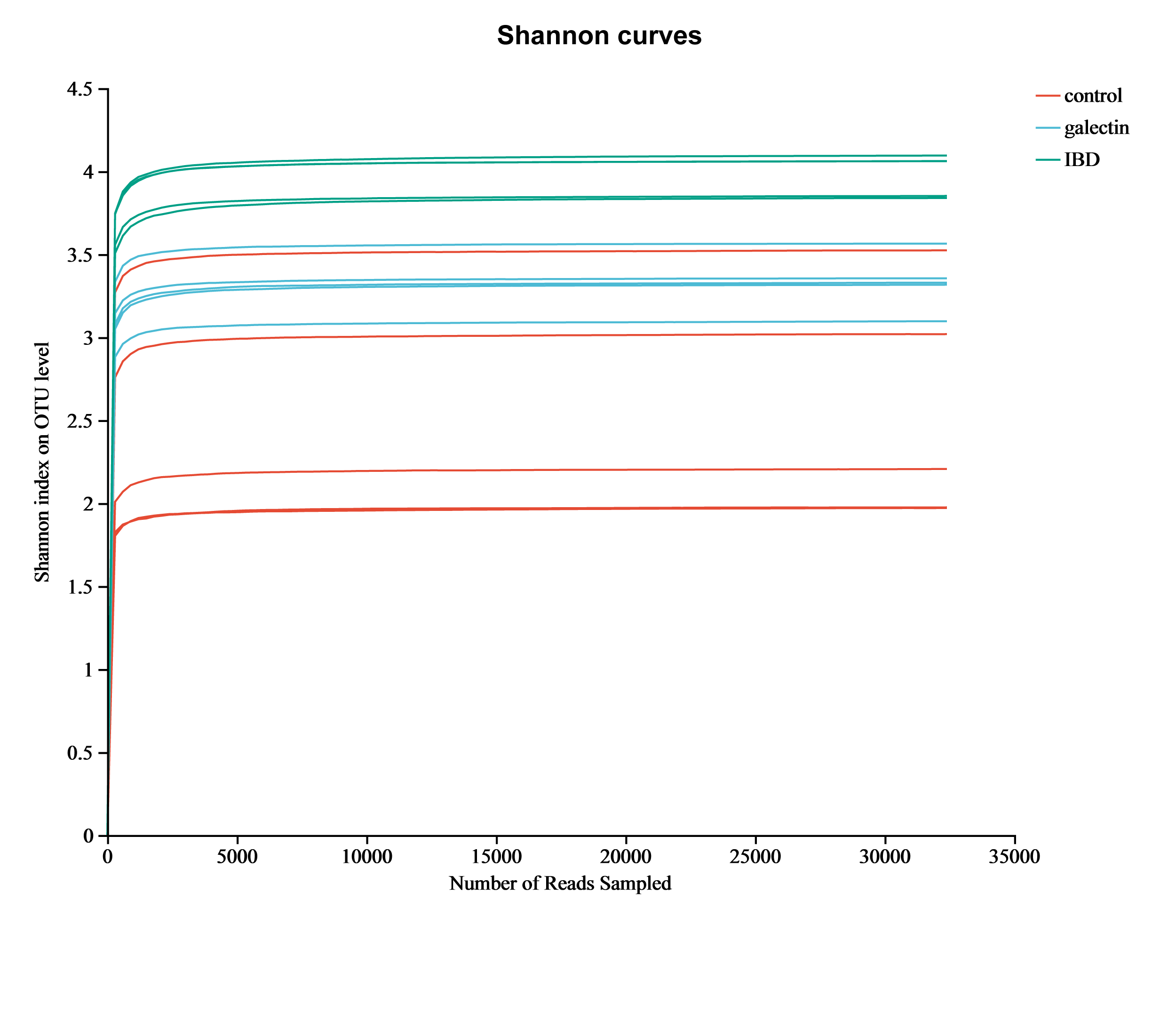

Supplement: Supplementary file 3 — Additional file 3. Rarefaction curves (Shannon indices) of all the samples. [file 13567_2023_1262_MOESM3_ESM.tif]

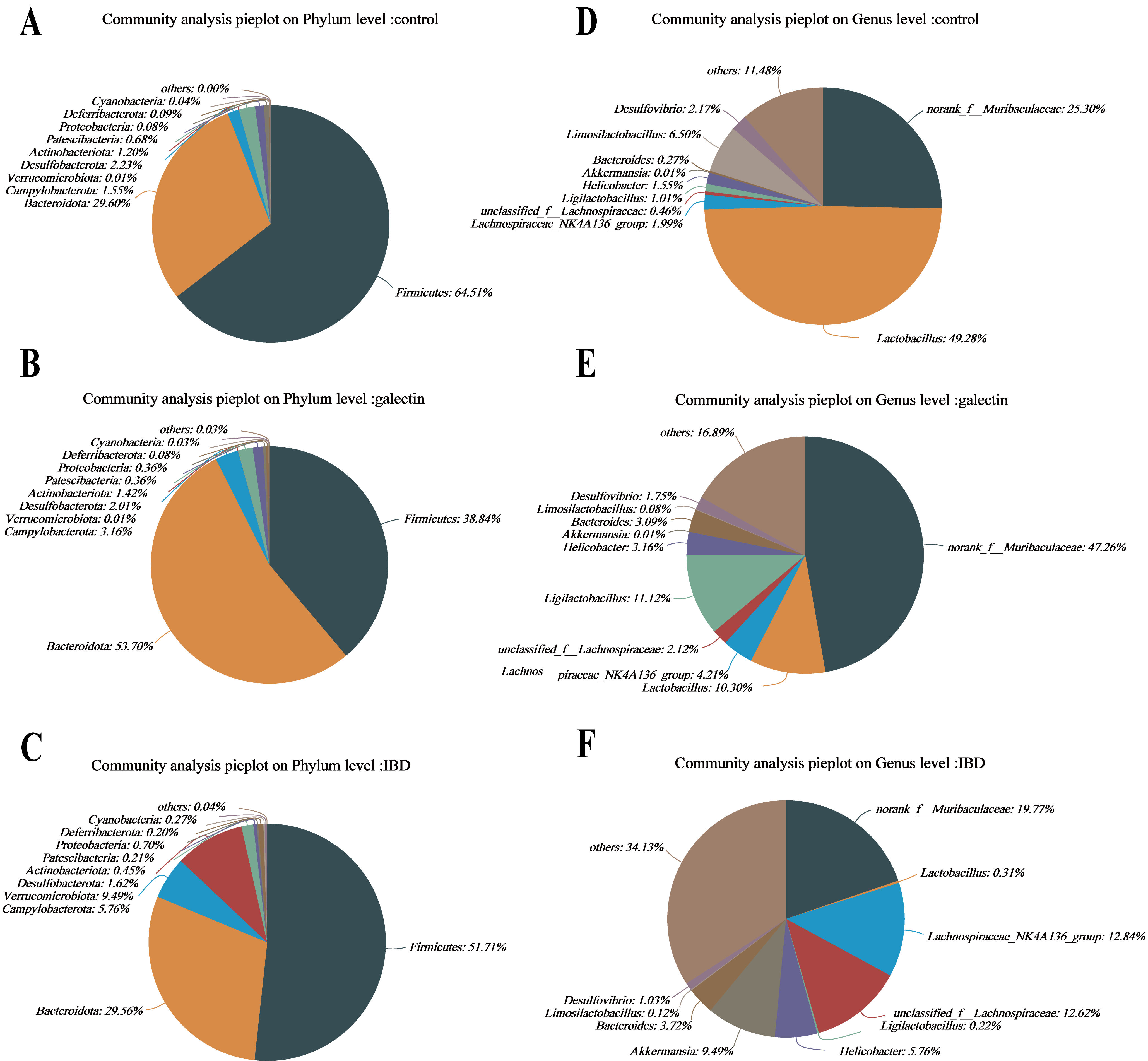

Supplement: Supplementary file 4 — Additional file 4. The detailed relative abundance of the bacterial community at the phylum and genus levels. (A) and (B) Control group; (C) and (D) Galectin group; (E) and (F) IBD group. [file 13567_2023_1262_MOESM4_ESM.tif]

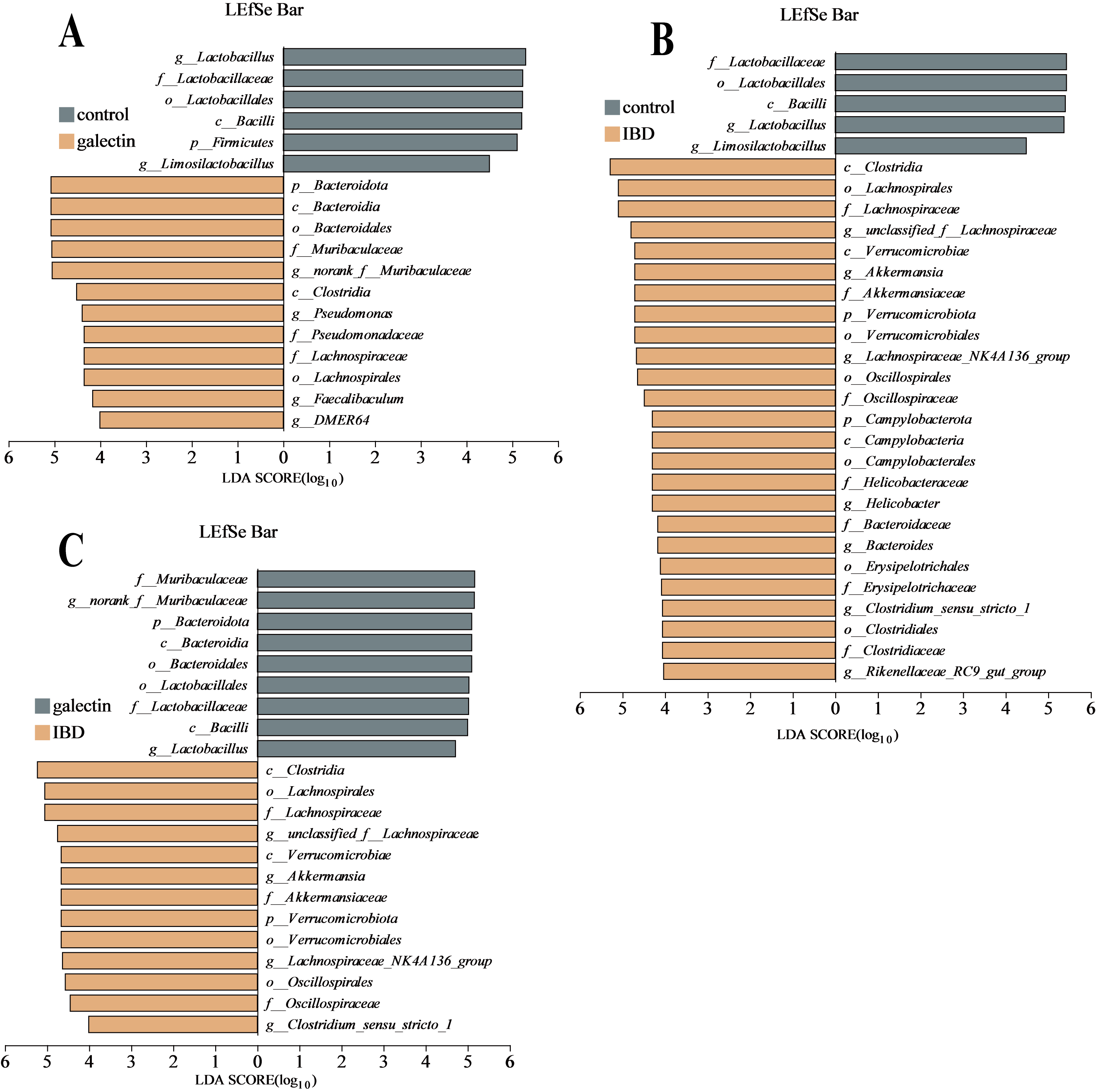

Supplement: Supplementary file 5 — Additional file 5. LefSe analysis of all significantly different genera between each group. Only genera with LDA scores >4.0 and P < 0.05 are listed in the figures. [file 13567_2023_1262_MOESM5_ESM.tif]
